# An In Vitro Evaluation of the Antibacterial Efficacy of Solanum xanthocarpum Extracts on Bacteria From Dental Plaque Biofilm

**DOI:** 10.7759/cureus.45202

**Published:** 2023-09-14

**Authors:** Deepavalli Arumuganainar, Gopinath Subramaniam, Arun Kurumathur Vasudevan, Balaji Subbusamy Kanakasabapathy

**Affiliations:** 1 Department of Periodontics, Ragas Dental College and Hospital, Chennai, IND; 2 Department of Pharmaceutics, Sri Ramachandra Institute of Higher Education and Research, Chennai, IND; 3 Department of Periodontics, Amrita School of Dentistry, Chennai, IND; 4 Department of Periodontology and Implantology, Sri Ramachandra Institute of Higher Education and Research, Chennai, IND

**Keywords:** minimum inhibitory concentration, solanum xanthocarpum, periodontal disease, herbal formulation, antimicrobial activity

## Abstract

Objective: The focus of research has recently shifted toward developing herbal-based medicines due to the emerging bacterial resistance and side effects of antibiotics. *Solanum xanthocarpum (Sx)* is a medicinal plant with potent pharmacological properties. This study aimed to evaluate the antibacterial activity of its crude extracts on bacteria isolated from dental plaque in patients with gingivitis.

Materials and methods: Aqueous, ethyl acetate, hexane, chloroform, and ethanolic extracts were prepared from *Sx*. Dental plaque samples were collected from patients with plaque-induced gingivitis. Disk diffusion assay was performed to determine the antibacterial activity of the extracts at concentrations of 25 mg/ml, 50 mg/ml, and 75 mg/ml with ampicillin 200 mg/ml as a positive control. The minimum inhibitory concentration (MIC) of the aqueous extract was also evaluated by broth dilution test against bacteria isolated from dental plaque biofilm.

Results: The antibacterial activity was estimated by measuring the zones of inhibition through the disc diffusion method. The Kruskal Wallis with Dunn post hoc test performed for intergroup comparison between the various extracts showed a statistically significant difference in inhibition of bacterial growth between 25 mg/ml and 75 mg/ml concentrations. There was no significant difference between the 75 mg/ml *Sx* concentration and the positive control. In addition, the MIC was elucidated to be 0.625 g/ml, at which there was maximum inhibition of bacterial growth.

Conclusion: The *Sx *extract exhibited antibacterial activity against periodontal pathogens. Thus, it can be concluded that optimum concentrations of *Sx *could be used in therapeutic strategies to prevent and manage periodontal diseases.

## Introduction

Periodontal disease is a multifactorial, chronic inflammatory disease of the periodontium resulting in progressive destruction of the tooth-supporting structures, eventually leading to tooth loss [1]. It is principally polymicrobial in origin which entails a synergistic and dysbiotic transformation of the oral microbiota which orchestrates a complex disease mechanism [2]. This in turn leads to the disruption of tissue homeostasis and host immune-inflammatory response and, eventually, periodontal destruction. It also influences the systemic inflammatory burden and may lead to the worsening of chronic inflammatory conditions such as diabetes mellitus, cardiovascular diseases, rheumatoid arthritis, and adverse pregnancy outcomes [3-6]. It also has a significant impact on the oral health-related quality of life because of oral malodor, bleeding from the gingiva, and mobility of teeth [7].

Mechanical removal of plaque biofilm by judicious tooth brushing and the use of several oral hygiene aids result in effective prophylaxis against periodontal diseases. However, a mainstream of population like aged individuals and physically or mentally challenged individuals find the process of mechanical plaque removal challenging and, thus, incomplete [8]. In addition, the oral cavity is considered a highly heterogeneous system containing distinct ecological niches which include supragingival tooth surfaces, periodontal pocket, tongue, buccal mucosa, the floor of the mouth, vestibule, palate, and tonsils [9]. These niches harbor polymicrobial communities which play a crucial role in maintaining oral health, and any imbalance may result in microbial shift or dysbiosis which in turn leads to initiation and progression of periodontal disease [10]. Studies have also reported translocations of periodontopathogens among these niches [9]. Hence, apart from the mechanical plaque removal process by tooth brushing and tongue cleaning, eradication or inhibition of the growth of microbial communities from the niches is of paramount importance. Antimicrobial oral rinses are largely employed for this purpose. However, reports have suggested that the prolonged use of oral rinses may result in undesirable side effects such as taste aberrations, tooth staining, and burning sensation in the oral cavity [11].

In addition, the poly-microbial etiology of the disease and complex clinical presentation make it a challenging task for the management of periodontal diseases. Chemotherapeutic agents have been used as adjuvants to professional mechanical debridement to hasten the healing process by administering either systemically or locally at the site of inflammation [12,13]. However, due to undesirable side effects such as the development of bacterial resistance, hypersensitivity, and gastric intolerance, much attention has been paid to developing newer antimicrobials that are derived from nature. Since phytochemicals have always inspired most clinically active drugs, the search naturally shifted toward plant-derived active principles and introduced contemporary methods for the prevention and management of periodontal diseases.

Each geographic zone in the world has its own traditional medical practices based on locally available flora. In this regard, knowledge derived from traditional wisdom and its documentation is of invaluable aid to us. Evidence from literature has also suggested that herbal derivatives can serve as suitable substitutes for synthetic agents in the prophylactic and curative care of periodontal diseases owing to their considerable natural effect, augmented safety, and reduced cost [14,15]. In this context, numerous herbs have been explored and are identified to possess antimicrobial properties [16,17]. During the last decades, the extracts of medicinal herbs possessing anti-bacterial, antioxidant, and anti-inflammatory activities have been utilized for the prevention and treatment of various oral diseases [18,19].

*Solanum xanthocarpum* (*Sx*) is a perennial herb belonging to the *Solanaceae* family and grown as a wild plant in various parts of India [20]. The fruits are edible and are consumed by the Irula tribes of Hasanur hills in Tamil Nadu [21] and by the Kuruma and Paniya tribes of Wayanad district in Kerala [22]. To date, more than 200 active metabolites have been identified such as alkaloids, flavonoids, glycoalkaloids, phenolic compounds, tannins, and terpenoids. Several reports have suggested a wide range of biological activities, namely, antimicrobial, hepatoprotective, anti-inflammatory and antithrombotic properties, anti-oxidant, anti-fungal, anti-allergic, immunomodulatory, anti-cancer, and hypoglycemic properties [23-28]. The fruits are considered a valuable herbal source for traditional therapists in the treatment of various conventional diseases in India [27]. Outcomes from non-clinical and clinical studies suggest that a wide range of formulations are being prepared from either the crude extracts or the isolated phytochemicals of *Sx*.

In the present study, the antimicrobial activity of the *Sx* crude extract derived from five different solvents was investigated against periodontopathogens isolated from the dental plaque biofilm in patients with gingivitis. This study also aimed to arrive at an optimal antimicrobial concentration of aqueous extract of *Sx* for use in various formulations for the prevention and management of periodontal diseases as well as other oral diseases.

## Materials and methods

Collection of raw materials and extraction

Fruiting material of *Sx* was collected in and around Chennai and Tirunelveli, Tamil Nadu, India. The plant was authenticated by a taxonomist, and a voucher specimen (No: PARC/2021/4483) was deposited at the Plant Anatomy Research Center, Chennai, Tamil Nadu, India, for future reference.

The collected parts were carefully washed and shade-dried for five to six days. The dried plant material was ground to a coarse powder using a blender and stored in airtight containers. Five different solvents were used for extraction, including distilled water, hexane, ethyl acetate, chloroform, and ethanol. Powdered samples weighing 300 g were subjected to Soxhlet extraction for seven hours, 20 cycles, using 5.0 liters of each solvent. The extracts were dried and concentrated by evaporating the solvent completely under a vacuum at the range of boiling points of solvents using a rotary evaporator until it gave a yield of 20%. Each preparation was filtered through a sterilized Whatman No.1 filter paper and finally concentrated to dryness under vacuum at 40°C using a rotary evaporator. The dried extract, thus obtained, was sterilized by overnight UV irradiation, checked for sterility on nutrient agar plates, and stored in sterile condition at 4°C until further use. The dried extract was weighed to determine the percent yield of each extract (Table 1). The percentage of extraction yield obtained was highest with ethanol (8.44%) followed by hexane (3.47%), chloroform (2.73%), ethyl acetate (2.52%), and aqueous (1.88%). The dried extracts were reconstituted to 10% in respective solvents to determine anti-bacterial activity.

**Table 1:** Percentage yield of the extracted materials of dried powdered fruits of Sx by different solvents

S No.	Solvent	Weight of plant powder taken (g)	Extract obtained (g)	Extraction yield (%)
1	Distilled water	300	5.63	1.88
2	Hexane	300	10.4	3.47
3	Ethyl acetate	300	7.57	2.52
4	Chloroform	300	8.2	2.73
5	Ethanol	300	25.31	8.44

Collection of plaque samples

Plaque samples were collected from patients who reported to the Department of Periodontics, Ragas Dental College and Hospital, Chennai, Tamil Nadu, India. The patients were 30 to 50 years old and were diagnosed with plaque-induced gingivitis. Clinical diagnosis was based on the American Academy of Periodontology Classification 1999 [29]. For periodontal diagnosis, a single calibrated periodontist recorded the plaque index, gingival index, and probing pocket depth at six sites per tooth. All patients were systemically healthy and exhibited ≥20 teeth, with at least a few exhibiting clinical signs of gingival inflammation. Patients giving a history of ongoing periodontal therapy, antibiotic intake in the preceding three months, smokers, alcoholics, and pregnant and lactating women were excluded. All patients gave written informed consent for the collection of plaque samples. The study was approved by the Institutional Ethics Committee of Ragas Dental College and Hospital (No: 20170762) and Sri Ramachandra Institute of Higher Education and Research (IEC/21/JUN/163/45), Chennai, Tamil Nadu, India.

Sampling procedure

The patients were positioned in the dental chair and were asked to rinse their mouths with water. The supragingival plaque samples were collected from sites exhibiting clinical signs of inflammation. The selected site was isolated with cotton rolls. The plaque was gently collected with a sterile curette, placed in Eppendorf tubes containing phosphate-buffered saline, and stored at -20°C until further analysis.

Obtaining bacterial colonies from plaque samples

Nutrient agar medium was prepared and autoclaved. The sterilized medium was poured into sterile petri plates and allowed to solidify. The plaque samples were serially diluted up to 10^-7^ dilutions separately. About 0.1 ml of the sample from 10^-4^ and 10^-7^ dilutions were inoculated in nutrient agar plates. The plates were incubated at 37°C for 24 hours to observe colonies. Individual colonies were observed at 10^-5^ concentrations (Table 2).

**Table 2:** Estimation of total bacterial count in each plaque sample

S No.	Plaque sample	Colonies observed at 10^-5 ^concentration	Total bacterial count
1	S1	300	43 X 10^ 5^
2	S2	300	17 x 10^5^
3	S3	300	25 x 10^5^
4	S4	300	23 x 10^5^
5	S5	300	87 x 10^5^
6	S6	300	40 x 10^5^
7	S7	300	81 x 10^5^

Determination of antibacterial activity of five different *Sx* extracts

Before the commencement of the procedure, the media and the glass wares were sterilized in an autoclave at 121°C at 15 lb/in2 pressure for 20 minutes, and the glass wares were then kept in a hot air oven at 100°C for two to three hours to remove the water droplets. Antimicrobial activity was tested in vitro using the disc diffusion method [30]. The sub-cultured strain was inoculated in 50 ml of nutrient broth and kept at 37ºC for 24 hours. Small inoculums were evenly swabbed using sterile cotton swabs on nutrient agar plates. Sterile 5 mm discs were prepared from Whatman No.1 filter paper, loaded with respective extracts, and allowed to dry at 37ºC for 20 minutes. For the crude extract, the concentrations used were 25 mg/ml, 50 mg/ml, 75 mg/ml, and 100 mg/ml. The antibacterial activity of *Sx* extracts was compared with standard ampicillin (200 mg/ml) as a control. Antibacterial activity was determined by measuring the zones of inhibition that formed around the disc. The experiment was performed in triplicate, and the mean diameter was calculated using the triplicate values.

Determination of minimum inhibitory concentration of the aqueous crude extract

The minimum inhibitory concentration (MIC) of aqueous crude extract was determined by the broth microdilution method given by the Clinical and Laboratory Standards Institute using 96-well microtiter plates (Singh et al., 2013) [31]. Twofold serial dilutions of the extract of concentrations 10, 5, 2.5, 1.25, 0.625, 0.3125, 0.1563, 0.0781, 0.0391, and 0.0195 g/mL were made using Mueller-Hinton broth. About 5 µl inoculum (5 × 105 CFU/mL) was added to each well, and the plates were incubated at 37°C for 24 hours. From the above assay, 100 µl of inoculum was taken from each well and spread onto MHA plates to validate the MIC assay. The lowest concentration, which completely inhibited the growth of microbes, was recorded as MIC of an aqueous extract of *Sx*.

Statistical analysis

Mean and standard deviation were used as descriptive statistics. Zone of inhibition produced by extracts from various solvents were tested for normality in data using the Shapiro-Wilk test. It was statistically significant and, hence, was considered not normally distributed. Accordingly, the Kruskal Wallis with Dunn post hoc test was performed for intergroup comparison. A p-value of <0.05 was considered statistically significant.

## Results

**Figure 1:**
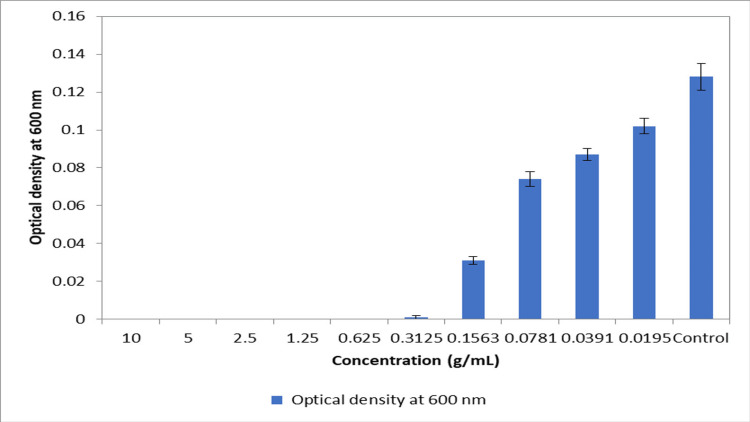
MIC of Sx crude aqueous extract (g/ml) against clinically isolated periodontal pathogens

**Figure 2:**
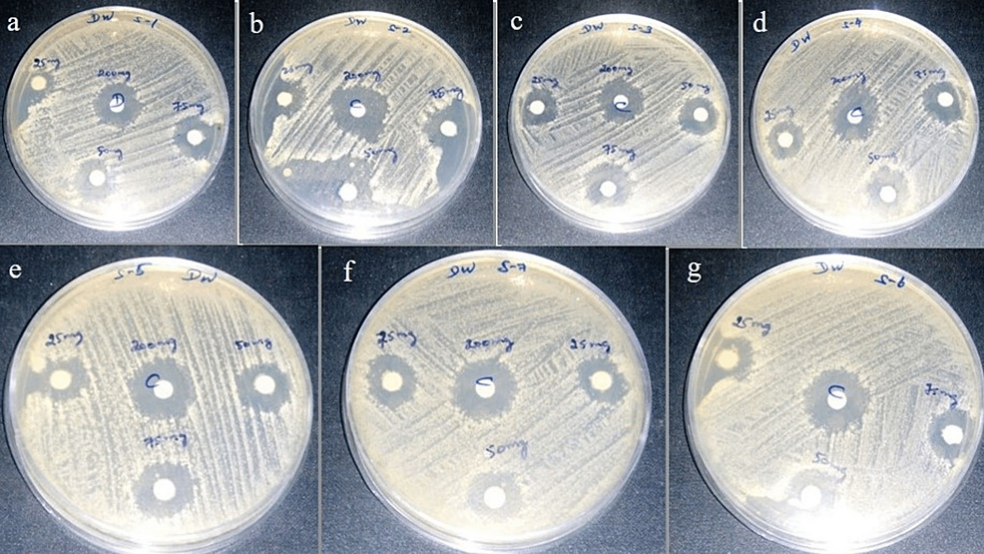
Antibacterial activity (zones of inhibition) of the aqueous extract of Sx on seven plaque samples (a-g)

The percentage yield of the extracted materials was calculated to find the efficiency of eluting solvents as shown in Table 1. It was found that maximum efficiency was shown by ethanol followed by hexane > chloroform > ethyl acetate > distilled water. Figure 1 shows the MIC of crude aqueous extract evaluated against bacteria isolated from dental plaque biofilm. As the concentration of 0.625 g/ml showed complete inhibition of bacterial growth, this was considered the MIC of the aqueous crude extract of *Sx*. The individual colonies formed in the nutrient agar plates after incubation were observed, and the total bacterial count was estimated in each plaque sample, as shown in Table 2. The antibacterial activity of *Sx* extracts was evaluated by the disk diffusion assay using sub-cultured organisms isolated from the dental plaque samples from seven patients with gingivitis. Table 3 shows the mean zones of inhibition produced by extracts of five different solvents of *Sx* using the disk diffusion assay. Figure 2 shows the zones of inhibition produced by the aqueous extract of various concentrations on the seven dental plaque samples. The Kruskal Wallis with Dunn post hoc test was performed for intergroup comparison of the antibacterial activity between the different concentrations of each extract, as shown in Table 4, where x1, x2, x3, and x4 denote the different concentrations of the extracts used for assessing the antibacterial activity (x1- 25 mg/ml concentration; x2- 50 mg/ml concentration; x3- 75 mg/ml concentration; x4- 200 mg/ml which is the control, ampicillin). With respect to the aqueous extract, there was a statistically significant difference between x1 and x3 and between x1 and x4. With respect to the hexane and ethyl acetate extracts, there was a significant difference between x1 and x3, x1 and x4, and x2 and x4. With respect to the chloroform extract, there was a significant difference between x1 and x3, x1 and x4, and x2 and x4. With respect to the ethanol extract, there was a significant difference between x1 and x4 and x2 and x4.

**Table 3:** Antibacterial activity (zones of inhibition) of the five different extracts at various concentrations against bacteria isolated from dental plaque biofilm Sx: Solanum xanthocarpum

Extract of *Sx*		Zones of inhibition in mm			Control (ampicillin)	p-value
		25 mg	50 mg	75 mg	200 mg	
Aqueous extract	Mean	12	13.42857	14.42857	13.57143	0.0101
	SD	1	1.397276	1.397276	0.786796	
	Min	11	12	13	13	
	Max	14	16	17	15	
Hexane extract	Mean	8	9.428571	11.14286	13.14286	0.002212
	SD	1.632993	2.070197	1.573592	2.340126	
	Min	6	7	9	10	
	Max	10	12	13	16	
Ethyl acetyl extract	Mean	10.71429	12	13.28571	14.28571	0.0198
	SD	2.984085	1.527525	1.496026	1.112697	
	Min	7	10	11	13	
	Max	15	14	15	16	
Chloroform extract	Mean	10.85714	12.28571	13.71429	14.42857	0.00009843
	SD	0.899735	0.755929	0.755929	0.9759	
	Min	10	11	13	13	
	Max	12	13	15	16	
Ethanol extract	Mean	11.14286	12	13	14.14286	0.0001135
	SD	0.899735	0.57735	0.57735	0.899735	
	Min	10	11	12	13	
	Max	12	13	14	15	

**Table 4:** Intergroup comparisons of the antibacterial activity among the different concentrations of each extract against bacteria isolated from dental plaque biofilm Where x1 - 25 mg/ml; x2 - 50 mg/ml concentration; x3 - 75 mg/ml concentration; x4 - 200 mg/ml - control, ampicillin

Extract	Pair compared	Mean rank difference	Z	SE	Critical value	p-value
Aqueous extract	x1-x2	-8.2857	1.9326	4.2873	11.3111	0.05328
	x1-x3	-14	3.2655	4.2873	11.3111	0.001093^*^
	x1-x4	-10	2.3325	4.2873	11.3111	0.01968^*^
	x2-x3	-5.7143	1.3328	4.2873	11.3111	0.1826
	x2-x4	-1.7143	0.3999	4.2873	11.3111	0.6893
	x3-x4	4	0.933	4.2873	11.3111	0.3508
Hexane extract	x1-x2	-4.5	1.0321	4.3601	11.5032	0.302
	x1-x3	-10.5714	2.4246	4.3601	11.5032	0.01533^*^
	x1-x4	-15.5	3.555	4.3601	11.5032	0.000378^*^
	x2-x3	-6.0714	1.3925	4.3601	11.5032	0.1638
	x2-x4	-11	2.5229	4.3601	11.5032	0.01164^*^
	x3-x4	-4.9286	1.1304	4.3601	11.5032	0.2583
Ethyl acetate	x1-x2	-4.5	1.0552	4.2644	11.2507	0.2913
	x1-x3	-12.2143	2.8642	4.2644	11.2507	0.00418^*^
	x1-x4	-17.8571	4.1875	4.2644	11.2507	2.82E-05
	x2-x3	-7.7143	1.809	4.2644	11.2507	0.07045
	x2-x4	-13.3571	3.1322	4.2644	11.2507	0.001735^*^
	x3-x4	-5.6429	1.3232	4.2644	11.2507	0.1858
Chloroform	x1-x2	-6.1429	1.4212	4.3223	11.4035	0.1553
	x1-x3	-14.5	3.3547	4.3223	11.4035	0.000795^*^
	x1-x4	-17.9286	4.1479	4.3223	11.4035	3.36E-05
	x2-x3	-8.3571	1.9335	4.3223	11.4035	0.05318
	x2-x4	-11.7857	2.7267	4.3223	11.4035	0.006397^*^
	x3-x4	-3.4286	0.7932	4.3223	11.4035	0.4276
Ethanol extract	x1-x2	-2.0714	0.4778	4.3352	11.4373	0.6328
	x1-x3	-7.6429	1.763	4.3352	11.4373	0.0779
	x1-x4	-12.2857	2.834	4.3352	11.4373	0.004597^*^
	x2-x3	-5.5714	1.2852	4.3352	11.4373	0.1987
	x2-x4	-10.2143	2.3561	4.3352	11.4373	0.01847^*^
	x3-x4	-4.6429	1.071	4.3352	11.4373	0.2842

## Discussion

Traditional antibiotics have shown insufficient efficacy in periodontal management and have raised fears of developing resistant microorganisms [32]. The primary objective of the present study was to evaluate the antibacterial effect of *Sx* on periodontopathic bacteria. *Sx* is one of the ancient medicinal herbs whose efficacy has been well-recognized in Indian folk and traditional medicines. However, its utilization is yet to reach the modern pharmaceutical form.

*Sx* is well recognized for its vast range of therapeutic activities. Evidence from the literature suggests that *Sx* fruits and seeds are a vital source of a large number of bioactive phytochemicals [33] with an extensive curative application for a wide range of human diseases and conditions, including respiratory, gastrointestinal, cardiac, cough, fever, asthma, etc. [28]. Findings from non-clinical and clinical studies also suggest that a wide range of formulations, such as powders, churnas, etc., are being prepared from either the crude extracts or the isolated phytochemicals of *Sx*, especially the fruits.

Hence, this study was intended to evaluate the anti-microbial activity against periodontopathogens and eventually formulate newer drugs incorporating its most active components for the treatment of periodontal diseases. Further, it is also presumed that the additional pharmacological activities, namely, the anti-oxidant [34] and anti-inflammatory activities [35], could supplement the anti-microbial activity in controlling the progression of periodontal disease.

In this study, five different extracts of *Sx* were initially obtained using solvents: aqueous, hexane, ethyl acetate, chloroform, and ethanol. All five extracts of *Sx* showed potent antibacterial activity against periodontopathogens. For each extract, the concentrations used were 25 mg/ml (x1), 50 mg/ml (x2), and 75 mg/ml (x3) with standard 200 mg/ml (x4) ampicillin as control.

The statistically significant difference in the antibacterial activity between the concentrations of 25 mg/ml and 75 mg/ml of the extracts, namely, aqueous, hexane, ethyl acetate, and chloroform, suggested that, as the concentration of the extract was increased, the antibacterial activity also increased. Concerning the ethanol extract, there was no significant increase between the concentrations of 25 mg/ml and 75 mg/ml, which suggested that the concentration gradient did not influence the antibacterial effect of the ethanol extract. With respect to all five extracts, there was a significant difference between the concentration of 25 mg/ml and the control ampicillin, making it evident that the control was more potent than the concentration of the extract at 25 mg/ml. However, there was no significant difference between the concentration of 75 mg/ml and the control drug, ampicillin, suggesting that the potency of the extracts with all solvents was similar to that of the control drug, ampicillin. Moreover, the larger zone of inhibition produced by the positive control could be attributed to the higher concentration used and its higher diffusibility in the agar media.

Abbas et al. [36] analyzed the antimicrobial properties of fruits of *Sx *against gram-positive bacteria (*Micrococcus varians*, *Micrococcus luteus*, and *Staphylococcus aureus*), gram-negative bacteria (*Salmonella typhi*, *Pasteurella multocida*, *Escherichia coli*, *Klebsiella pneumoniae*, *Vibrio cholerae*), and fungi (*Aspergillus niger*, *Aspergillus flavus*, *Aspergillus fumigatus*) and have found that methanolic extract had the maximum activity. They have suggested that future research should focus on studying antimicrobial activity on other pathologic organisms. Udayakumar et al. [37] evaluated the antimicrobial activity of various parts of *Sx *against various species of bacteria and demonstrated high sensitivity to *Klebsiella pneumoniae* and *Salmonella typhi*. Further, moderate sensitivity to *Escherichia coli* and lower sensitivity to *Bacillus cereus* were observed. Previous studies have reported antibacterial activity against selective organisms. However, in this study, the activity was evaluated against clinically isolated organisms from plaque biofilm because most of these are anaerobic and are communally responsible for the causation of dysbiosis in oral ecological niches leading to periodontal disease initiation and progression. In addition, with over 700 species of organisms present in the oral cavity, most of them being involved in the pathogenesis of the disease, it is prudent to elucidate the antibacterial activity against the entire microbiome instead of a single species of organisms.

In this study, even though all the extracts showed potent anti-bacterial activity, the aqueous extract was chosen for further experimentation and analyses so that the final product does not leave behind any traces of toxic substances. The MIC of the aqueous crude extract was evaluated against clinically isolated periodontal pathogens using the broth dilution method. The studied concentrations showed a clear increase in antibacterial activity with an increase in concentration. There was complete inhibition of bacterial growth at the concentration of 0.625 g/ml. Hence, this concentration was considered the MIC of the aqueous crude extract of *Sx*. Several other studies have evaluated the MIC of *Sx* against an array of pathogens. A report by Natarajan et al. demonstrated the MIC of chloroform, methanol, and ethanol extracts of *Sx *to be 1.03 mg/ml, 0.06 mg/ml, and 1.01 mg/ml, respectively [38]. Another study by Pungle et al. reported that the MIC of *Sx*-capped silver nanoparticles ranged from 2.5 mg/ml to 5 mg/ml [39]. By and large, the findings of the present study suggest that *Sx* has potent antimicrobial activity against bacteria present in plaque. However, it should be remembered that the results pertain only to bacteria that could grow on the culture medium used in the study. In addition, there was a concentration-dependent gradient in the antimicrobial activity of aqueous extract, suggesting that it may prove a putative antimicrobial agent in the prophylactic and therapeutic strategies in periodontal management. Hence, efficient research on this herb can blend traditional knowledge with the current experimental methodology for developing formulations for various oral diseases.

Limitation

The limitation of our study is the small sample size of dental plaque used for evaluating the antibacterial activity of the five different extracts of *Sx*. Further studies with larger sample sizes and assays involving isolated bioactive compounds of the extracts may provide a robust foundation for the translation of the results into clinical trials involving drug formulations for periodontal therapy.

## Conclusions

This study delivers new information about the antibacterial effects of *Sx* fruit extracts derived from five different solvents. The results suggest that *Sx* extract might be the origin of active compounds that might be utilized for the management of periodontal diseases. The search can further be drawn to the next phase of drug development. Further research has to be carried out to isolate the potential bioactive compounds and elucidate their mechanism of action for treating a wide range of periodontal/oral diseases.
